# Stress monitoring using wearable sensors: IoT techniques in medical field

**DOI:** 10.1007/s00521-023-08681-z

**Published:** 2023-06-02

**Authors:** Fatma M. Talaat, Rana Mohamed El-Balka

**Affiliations:** 1 Faculty of Artificial Intelligence, Kafrelsheikh University, Kafrelsheikh, Egypt; 2 Computers and Control Systems Engineering Department, Faculty of Engineering, Mansoura University, Mansoura, Egypt

**Keywords:** Stress monitoring, Wearable sensors, Medical field, IoT

## Abstract

The concept “Internet of Things” (IoT), which facilitates communication between linked devices, is relatively new. It refers to the next generation of the Internet. IoT supports healthcare and is essential to numerous applications for tracking medical services. By examining the pattern of observed parameters, the type of the disease can be anticipated. For people with a range of diseases, health professionals and technicians have developed an excellent system that employs commonly utilized techniques like wearable technology, wireless channels, and other remote equipment to give low-cost healthcare monitoring. Whether put in living areas or worn on the body, network-related sensors gather detailed data to evaluate the patient's physical and mental health. The main objective of this study is to examine the current e-health monitoring system using integrated systems. Automatically providing patients with a prescription based on their status is the main goal of the e-health monitoring system. The doctor can keep an eye on the patient's health without having to communicate with them. The purpose of the study is to examine how IoT technologies are applied in the medical industry and how they help to raise the bar of healthcare delivered by healthcare institutions. The study will also include the uses of IoT in the medical area, the degree to which it is used to enhance conventional practices in various health fields, and the degree to which IoT may raise the standard of healthcare services. The main contributions in this paper are as follows: (1) importing signals from wearable devices, extracting signals from non-signals, performing peak enhancement; (2) processing and analyzing the incoming signals; (3) proposing a new stress monitoring algorithm (SMA) using wearable sensors; (4) comparing between various ML algorithms; (5) the proposed stress monitoring algorithm (SMA) is composed of four main phases: (a) data acquisition phase, (b) data and signal processing phase, (c) prediction phase, and (d) model performance evaluation phase; and (6) grid search is used to find the optimal values for hyperparameters of SVM (C and gamma). From the findings, it is shown that random forest is best suited for this classification, with decision tree and XGBoost following closely behind.

## Introduction

The Internet of things (IoT) has transformed the healthcare industry by enabling remote monitoring of patients' health status [1]. The integration of wearable sensors with IoT technologies has enabled continuous monitoring of physiological signals, leading to early detection of health problems and the ability to intervene before the condition worsens. Stress is a common health problem that can lead to various physical and mental disorders. IoT has the potential to be utilized in numerous industries, including efficient energy, transportation, agriculture, university campus connectivity, healthcare, logistics, and others, allowing physical objects to communicate and share information with one another through the internet [1].

IoT technology and machine learning (ML) have significantly impacted healthcare by shifting routine medical tests and healthcare services from hospitals to homes, making it easier for patients and healthcare professionals to utilize medical equipment [2]. With IoT integration, portable sensors can provide more precise data, and medical devices' usability can be enhanced by implementing an android program in conjunction with IoT. The implementation of various technologies, particularly IoT, is likely to have a significant impact on all sectors, particularly in the medical field, as it can improve people's quality of life [2].

IoT and ML have various applications in healthcare and daily life, with the internet's rapid growth decreasing the use of traditional patient service methods and increasing the use of electronic healthcare systems, providing patients and healthcare professionals with access to advanced medical equipment through IoT technology. ML and IoT devices are beneficial in various categories, such as remote monitoring and mechanical automation, offering convenience, cost savings, and increased patient satisfaction in medical care applications [3].

In the IoT healthcare system, a sensor can be identified as a “thing” based on three key characteristics. Firstly, it should be able to recognize and gather environmental data such as temperature, light, and precipitation, as well as monitor pulse-related functions like electrocardiogram, blood glucose levels, and blood oxygen levels. Secondly, it should be capable of transmitting data autonomously to a centralized controller, either dynamically or through another system. Lastly, it should be able to be inactive after the operation is completed, alerting medical professionals to take immediate action if necessary [1, 4]. Additionally, DNA origami has emerged as versatile nanomachines for transportation, sensing, and computing in two-dimensional patterns, and three-dimensional assembly [5–7].

Researchers have conducted innovative studies to improve healthcare systems by offering various analytical applications for managing data sources, including electronic health records (EHRs) and medical images [8]. While the development of apps and services in healthcare is tailored to user needs, it is clear that services are designed based on what the developer has to offer. Recently, various ML techniques have been employed such as convolutional neural networks [9, 10] in multiple applications across various fields, such as efficiently grading alcohol dependence, estimating accident severity in severe injuries, and identifying emotions in functional technologies [11, 12].

In conclusion, IoT and ML have brought about significant advancements in healthcare and daily life. With the integration of IoT, wearable sensors, and ML, healthcare professionals can obtain real-time data on patients' health status, enabling early detection and intervention before the condition worsens. The convenience, cost savings, and increased patient satisfaction of IoT devices have attracted attention in medical care applications, and the use of integrated technologies has the potential to improve electronic information management services, controlled communications, and system processing in various ways [2].

Stress is a common health problem that can lead to various physical and mental disorders. However, the integration of wearable sensors with IoT technologies has allowed for continuous monitoring of physiological signals, leading to early detection of stress-related problems and the ability to intervene before the condition worsens [13].

The use of IoT and ML in stress monitoring has gained attention in recent years. For example, a study proposed a wearable sensor system that combines physiological measurements, such as heart rate variability and skin conductance, with contextual data, such as location and activity level, to detect stress and track its progression [14]. This system utilized ML algorithms to analyze the data collected by the wearable sensors and provide personalized feedback and intervention to the user.

Other studies have explored the use of various sensors in stress monitoring, such as electroencephalography (EEG) and electromyography (EMG) sensors [15, 16]. These sensors measure brain activity and muscle tension, respectively, and can provide valuable information for stress monitoring and intervention.

The integration of IoT and ML in stress monitoring has the potential to improve the accuracy and efficiency of stress detection and intervention. By utilizing ML algorithms, the collected data can be analyzed in real time, allowing for early detection and intervention before the stress condition worsens. This can have a significant impact on the individual's quality of life and overall health.

In conclusion, stress monitoring by IoT and ML has the potential to revolutionize the healthcare industry by allowing for continuous monitoring of physiological signals and providing personalized feedback and intervention to the user. With advancements in wearable sensor technology and ML algorithms, the accuracy and efficiency of stress detection and intervention are likely to improve in the future.

Research gap:

- Limited studies have explored the integration of IoT and ML in stress monitoring, despite the potential benefits it offers.
- Few studies have focused on the use of multiple sensors for stress monitoring, with most research focusing on a single type of sensor.
- There is a need for further research to develop more accurate and efficient ML algorithms for stress detection and intervention.

Main contributions:

- The integration of wearable sensors with IoT and ML technologies can provide real-time stress monitoring and personalized feedback and intervention to the user.
- The use of multiple sensors, such as EEG and EMG, can provide valuable information for stress monitoring and intervention.
- ML algorithms can analyze the collected data in real time, allowing for early detection and intervention before the stress condition worsens, thus improving the individual's quality of life and overall health.

The structure of the remaining work is outlined as follows: Sect. 2 discusses recent research related to IoT in healthcare systems, Sect. 3 presents the proposed framework, Sect. 4 provides details of the experimental evaluation, and Sect. 5 concludes the effort.

## Related work

This section presents a discussion of various relevant research areas, including (1) human behavior recognition applications, (2) RL techniques for Internet of things (IoT), (3) wearable technology for stress monitoring and detection, and (4) challenges in physiological stress monitoring.

### Human behavior recognition applications

In the field of Human behavior recognition applications, there have been several studies. Zhao et al. [17] proposed a novel Res-Bidir-LSTM network to tackle HAR problems. This approach has shown high precision in the early stages of training, even though it takes a long time to develop. The Res-Bidir-LSTM method can be applied to challenging, large-scale HAR issues when sensor fusion is required. The HAR's input should include time series through the basic structure of the LSTM. One of the benefits of this technique is that it successfully solves the gradient vanishing problem.

Chang et al. [18] introduced a smart system, known as ST-Med-Box, based on deep learning techniques to identify medications. This system may help patients with chronic diseases to take numerous medications accurately and ensure they take the medication. Patients can download the available Android app and scan the QR codes on their drug packets to store the relevant medication information. Then, they can be reminded to take their medication.

### RL techniques for internet of things (IoT)

To find the optimum decision-making approach for the Internet of Things (IoT), a number of RL techniques have been investigated [19]. These include Q-learning, SARSA, Expected SARSA, and Monte Carlo. By utilizing RL techniques, the fog node's idle time may be minimized and its use of resources and services can be increased. In [20–22], a trustworthy cloud management system is proposed using RL.

### Wearable technology for stress monitoring and detection

The proposed study is influenced by a number of ongoing studies in the area of wearable technology for stress monitoring and detection. The RespiBAN and Empatica E4 wearables were used to construct the WESAD (Wearable Stress and Affect Detection) dataset [23]. The SWELL-KW dataset [24] combined computer logging, video (for facial expression), and Kinect (a 3D body posture sensor) to track the stress levels of 25 people while they worked on conventional knowledge tasks (doing a presentation, reading, producing reports, and sending emails) under time constraints.

The Affective-Road dataset [25] used Zephyr BioHarness 3.0 and Empatica E4 to measure stress levels throughout 10 drives while they were under various driving circumstances. Healey et al. [26] created a wearable glove with a photoplethysmogram (PPG) sensor inserted to track the levels of stress in 10 drivers as they traveled various routes. Similarly, Shi et al. [27] created a multi-node stress monitoring system based on ECG, EDA, and PPG data, and Muaremi et al. [28] used a smartphone and a Wahoo wearable breast belt to identify various levels of stress. A multi-sensor dataset of nurses who were employed by the hospital during the COVID-19 outbreak was produced by Hosseini et al. [29].

### Challenges in physiological stress monitoring

The literature study leads to the conclusion that there is still some debate over the best measuring strategy for physiological stress monitoring. The classification accuracy obtained by several research, albeit using the same physiological variables and classifiers, varied greatly. In the research, it is also unclear how sensitive and specific stress-related bio physiological measures like heart rate and respiration rate are [30, 31]. Table 1 presents the models that are commonly utilized in stress prediction, including random forest, XGBoost, and decision tree.

**Table 1:** Comparative analysis of the prevalent models employed in the field

Model	Algorithm description	Pros	Cons
Random forest	Random forest is a decision tree-based ensemble learning algorithm that builds multiple decision trees and aggregates their predictions to improve accuracy and reduce overfitting. Each tree in the forest is trained on a randomly selected subset of features and data samples. Random forest is widely used in various applications such as finance, marketing, and healthcare	Can handle high dimensional datasets with a large number of features Can handle missing values and categorical variables without requiring preprocessing Can provide feature importance rankings that can be useful for feature selection Has low bias and high variance, which can be beneficial for complex problems	May not perform well on small datasets with limited samples Can be slow and computationally expensive compared to other algorithms May overfit on noisy datasets
XGBoost	XGBoost (Extreme Gradient Boosting) is a boosting algorithm that trains multiple weak learners in a sequential manner, where each subsequent learner tries to correct the errors of the previous one. XGBoost is known for its scalability, efficiency, and high performance, and has been used in various fields such as natural language processing, image classification, and financial forecasting	Can handle missing values and categorical variables without requiring preprocessing Has a faster training time and better performance than other boosting algorithms Can handle imbalanced datasets and produce accurate results with a small number of samples Provides feature importance rankings that can be useful for feature selection	Can be sensitive to the choice of hyperparameters May overfit on small datasets with limited samples Can require more memory compared to other algorithms
Decision tree	A decision tree algorithm creates a treelike model of decisions and their possible consequences. It is a simple and interpretable algorithm that can handle both categorical and continuous data. Each node in the tree represents a feature, and each branch represents a decision rule based on the feature's value. Decision trees are used in various applications such as fraud detection, credit risk assessment, and medical diagnosis	Easy to understand and interpret Can handle both categorical and continuous data Can provide feature importance rankings that can be useful for feature selection Can handle missing values without requiring preprocessing	Easy to understand and interpret Can handle both categorical and continuous data Can provide feature importance rankings that can be useful for feature selection Can handle missing values without requiring preprocessing

Table 1 provides a comparative analysis of the commonly used models for stress prediction, namely random forest, XGBoost, and decision tree. Each algorithm is described in detail, highlighting its advantages and limitations. This table serves as a helpful reference for researchers and practitioners who are looking for the most suitable algorithm for their stress prediction task. By comparing the pros and cons of each algorithm, users can make an informed decision about which algorithm to use based on the specific requirements of their application.

Research gaps can be summarized in the following points:

- Limited amount of data points for deep LSTM neural network in human behavior recognition applications.
- Lack of comprehensive statistical analysis of the dataset in stress monitoring studies.
- Variability in classification accuracy for stress-related physiological measures across different studies.
- Small sample sizes in existing stress monitoring datasets.
- Limited comparison of different machine learning models for stress prediction.

## Stress monitoring algorithm (SMA) using wearable sensors

**Fig. 1:**
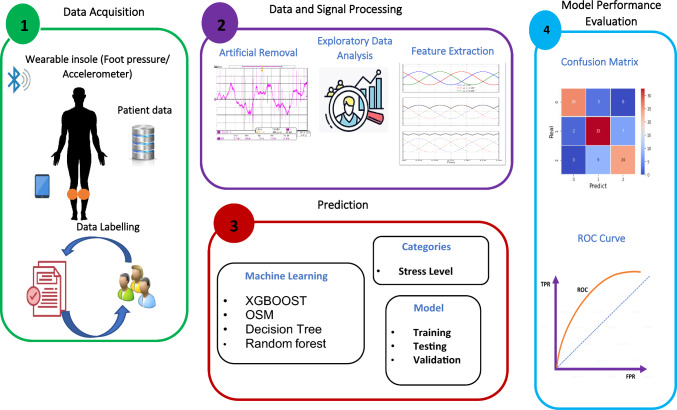
Architecture of the proposed stress monitoring using wearable sensors

The wearable sensors that are part of the suggested stress monitoring concept are simple for everyone to use. The proposed stress monitoring algorithm (SMA) is composed of four main phases as depicted in Fig. 1: (1) data acquisition phase, (2) data and signal processing phase, (3) prediction phase, and (4) model performance evaluation phase.

The proposed stress monitoring algorithm (SMA) using wearable sensors is a four-phase algorithm that aims to monitor and predict stress levels in individuals. Here is a brief overview of each phase:

1. *Data Acquisition Phase* In this phase, data are collected from the wearable sensors, which include physiological signals such as heart rate variability (HRV), skin conductance, and temperature. The sensors are worn by the individual throughout the day, and the data are collected continuously.
2. *Data and Signal Processing Phase* The data collected from the sensors undergo several processing steps to extract the relevant features that are indicative of stress. This phase includes preprocessing of the data, feature extraction, feature selection, and normalization.
3. *Prediction Phase* Once the relevant features have been extracted, the next step is to use machine learning algorithms to predict the stress levels of the individual. The machine learning algorithms used for this phase include decision trees, support vector machines, and artificial neural networks.
4. *Model Performance Evaluation Phase* In the final phase, the performance of the model is evaluated. The model is tested on a separate dataset to assess its accuracy in predicting stress levels. The performance of the model is evaluated based on various metrics.

### Data acquisition

The proposed architecture aims to benefit from wearable technologies created for real-time monitoring of physiological parameters related to stress. (1) Electrocardiogram (ECG), (2) infrared sensors, (3) hypertension monitoring, and (4) smoking behavior analysis are a few examples of wearable-based technologies.

#### Electrocardiogram (ECG) for heart rate monitoring

**Fig. 2:**
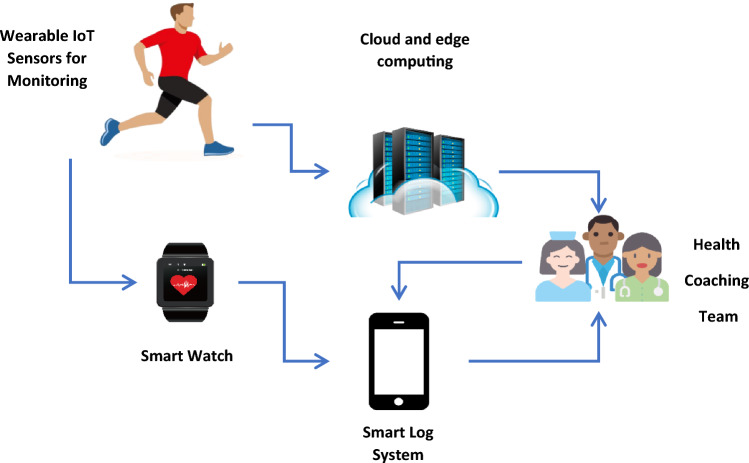
Using IoT architecture in health monitoring systems

These sensors will provide real-time heart rate monitoring using an ECG sensor to support the suggested model [32, 33]. Additionally, an IoT infrastructure with a prognosis algorithm is used to more efficiently notify doctors and patients' loved ones. A strategy for predicting the highs and lows of glucose levels was suggested [34, 35]. Figure 2 depicts this architecture.

#### Infrared sensors for blood glucose monitoring

Using near-infrared sensors, a portable noninvasive blood glucose monitoring system was created. This sensor, which is implanted at the fingertip to opportunistically measure blood sugar levels, computes the blood glucose concentrations. The signal is then increased and filtered before going to the microcontroller. The patient's glucose level was predicted using the voltages that were measured and the glucose information that would be shown on the LCD and transferred through Wi-Fi to a mobile device.

#### Hypertension monitoring

One of the factors that affects patients' chances of having a stroke is hypertension. Thus, one of the variables that must be carefully tracked in order to follow patients' conditions and make the best decision in order to avoid stroke is hypertension. For monitoring hypertension, it has previously been recommended to look at the blood pressure, the diagnosis, and the results in a hypertension patient setting [36].

#### Smoking behavior analysis

Wearable sensors have excelled at accurately identifying smoking events and individual puffs. The precise analysis of a person's smoking behavior requires the employment of these sensing techniques over the course of several weeks or months [37]. These sensors can help with the suggested model for tracking the smokers.

### Data and signal processing

The concept “digital signal processing” (DSP) refers to the application of digital processing to a range of signal processing tasks, such as those carried out by computer systems or more specialized digital signal processors. The digital signals examined in this manner are a set of values that represent samples of a continuous variable in a domain such as time, space, or frequency. In digital electronics, a pulse train—which is often generated by transistor switching—represents a digital signal. Algorithm 1 illustrates the DSP algorithm's steps. DSP consists of four main steps: (1) *Step 1* preprocessing of data; the first step is to preprocess the data collected by the wearable sensors. This includes filtering the data to remove any noise or artifacts, normalizing the data to account for any variations in sensor readings, and segmenting the data into windows of a fixed length for analysis.

(2) *Step 2* Feature Extraction: the second step involves extracting features from the preprocessed data. The features are statistical measures that describe the characteristics of the data, such as mean, variance, skewness, and kurtosis. These features are used as input to the prediction model in the next phase. (3) *Step 3* Feature Selection: in this step, a subset of the extracted features is selected for use in the prediction model. This is done to reduce the dimensionality of the data and improve the accuracy of the prediction model. Various feature selection techniques can be used for this purpose, such as principal component analysis (PCA) or correlation-based feature selection. (4) *Step 4* Signal Processing: this step involves applying signal processing techniques to the preprocessed data. These techniques include spectral analysis, time–frequency analysis, and wavelet analysis. The goal of this step is to extract more information from the data that can be used in the prediction model.
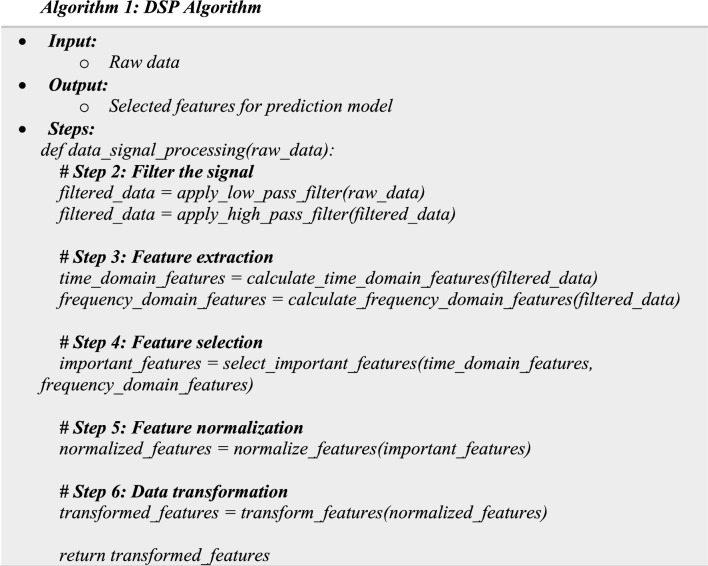


### Prediction

In this phase, a set of selected models are trained and tested and then the model with the highest performance is selected according to the steps illustrated in Algorithm 2. The prediction phase combines five main steps: (1) *Step 1* Preprocessing: Preprocess the data obtained from the previous phase, data and signal processing, by removing any noise or artifacts and normalizing the data. (2) *Step 2* Feature Extraction: Extract relevant features from the preprocessed data, such as heart rate variability, skin temperature, and respiration rate. (3) *Step 3* Model Selection: Select a set of models to train and test on the extracted features. Examples of models that can be used include support vector machines (SVM), random forests, and neural networks. (4) *Step 4* Training and Testing: (a) Train each selected model on a portion of the extracted feature data and test the trained model on the remaining portion of the data. (b) Use metrics such as accuracy, sensitivity, and specificity to evaluate the performance of each model.

(5) *Step 5* Model Performance Evaluation: (a) select the model with the highest performance based on the evaluation metrics obtained in the previous step. (b) Evaluate the selected model using cross-validation techniques to ensure that it is not overfitting the data.
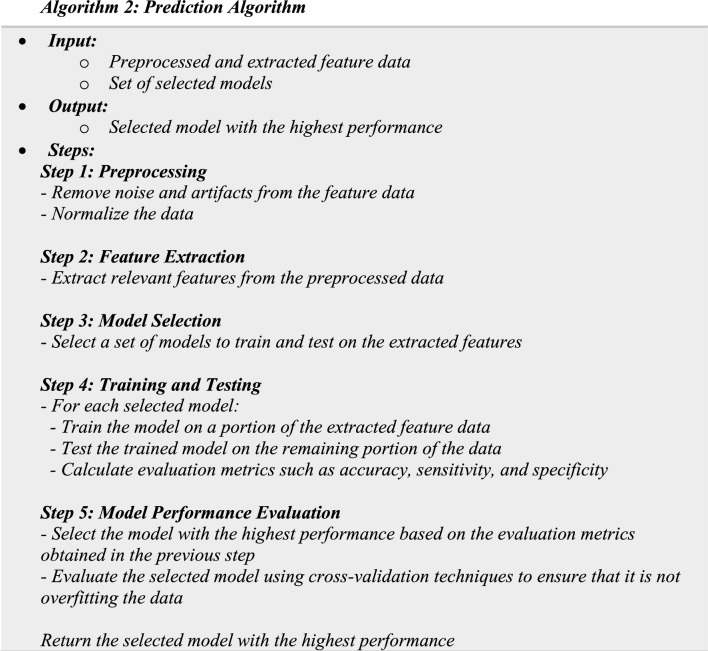


The prediction phase compares four ML algorithms: (1) XGBoost, (2) OSM, (3) decision tree, and (4) random forest.

1. XGBoost

**Fig. 3:**
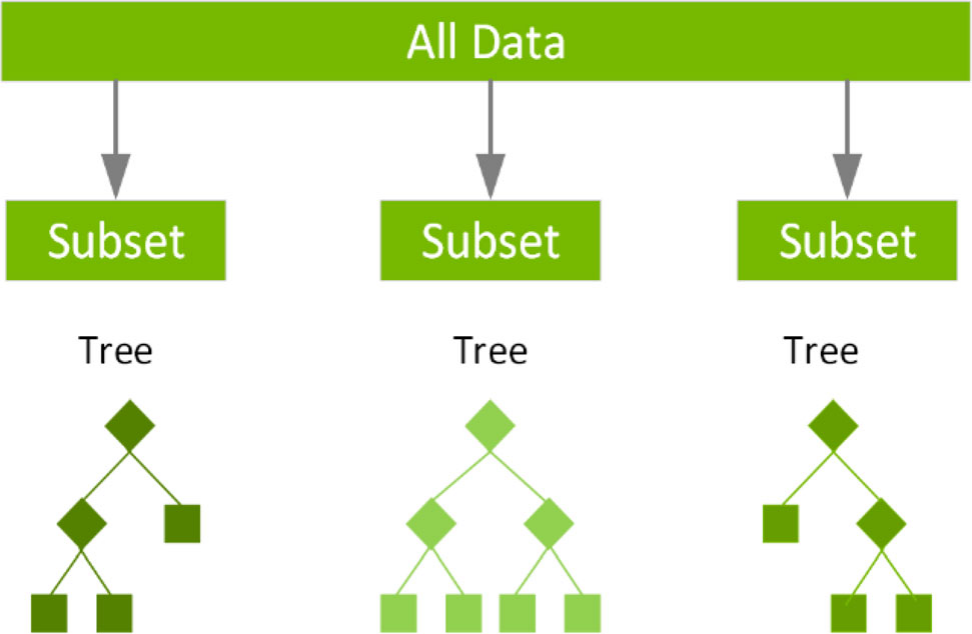
General architecture for XGBoost algorithm

**Fig. 4:**
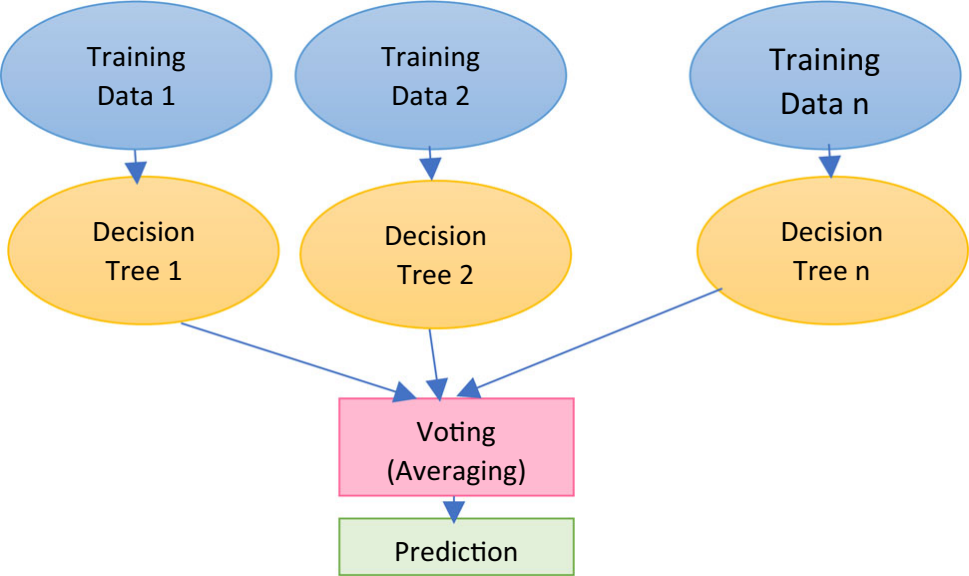
General architecture of random forest algorithm

Extreme gradient boosting model XGBoost is a sequential ensemble model. This is a gradient boost extension. The iterative XGBoost decision tree method employs a variety of decision trees. Each tree eventually picks up knowledge from the leftovers of every other tree. As opposed to accepting the final output of random forest that received the majority of votes, as illustrated in Eq. (1), the predicted output of XGBoost is the sum of all the results.

1 $$y_{i} = \mathop \sum \limits_{k = 1}^{n} f_{k} \left( {x_{i} } \right),f_{k} \in F$$

where $F$ is the set of regression trees, $f_{k}$ denotes a tree, and $y_{i}$ denotes the projected value for the *i*th occurrence of $x_{i}$. Figure 3 shows a general architecture for XGBoost algorithm. Figure 4 depicts the general architecture of random forest.

2. Optimized support vector machine (OSM)

**Fig. 5:**
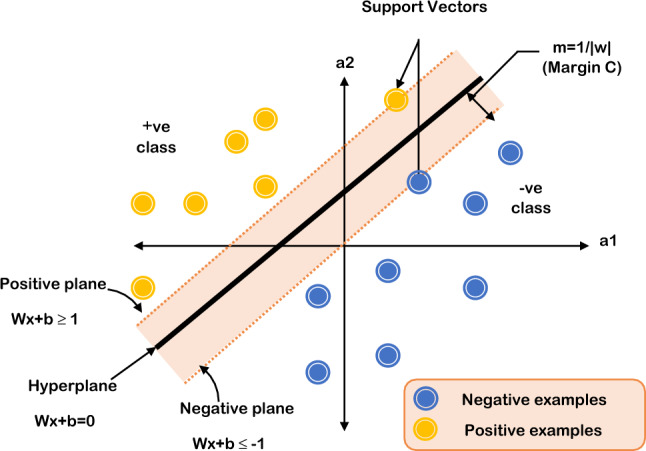
Separated data by a hyperplane defined by several support vectors


This stage outlines the optimization procedure used for the support vector machine hyperparameters (SVM). The best values for the SVM's hyperparameters (C and gamma) are found via grid search [38]. SVMs may effectively carry out nonlinear classification in addition to linear classification utilizing the kernel method, as shown in Fig. 5. SVM uses a number of kernel functions to get the best solution. The type of the kernel function and the trade-off constant C must be specified in order to implement SVMs. The soft margin's parameter C, which regulates how each support vector's influence is felt, is used. Algorithm 3 illustrates the total steps of the hyperparameters optimization algorithm.

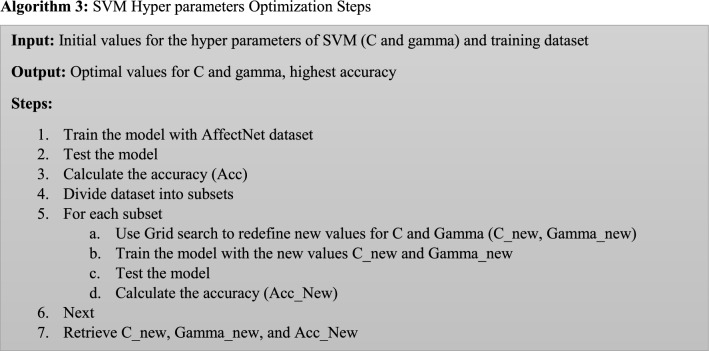

3. Decision tree

The decision tree algorithm is a type of supervised learning approach. The decision tree technique can handle both classification and regression issues, unlike other supervised learning methods. Using simple decision rules discovered from prior data, a training model is created with the use of a decision tree that may be used to forecast the class or value of the target variable (training data).

4. Random forest repressors

Random forest [39] uses the ensemble learning method for classification and regression as a supervised learning algorithm. It is a set of classification or regression trees that have not been trimmed that were produced using a random sample of training data. The qualities picked at random during the induction process. The prediction is created by combining the ensemble's guesses. Figure 4 displays the overall random forest architecture.

## Implementation and evaluation

This section introduces the used dataset and the performance evaluation.

### Human stress detection dataset

Titles for the Stress-Lysis.csv file [40] include “Humidity—Temperature—Step count—Stress levels.” The stress levels of the human being are here identified and assessed based on physical activity. Human body humidity, body temperature, and number of steps done by the user are all included in a collection of 2001 samples. Low stress, typical stress, and high stress are three different categories of stress that are evaluated.

### Stress level detection

A sample of dataset is depicted in Table 2.

**Table 2:** A sample of the used dataset

Humidity	Temperature	Step count	Stress level
21.33	90.33	123	1
21.41	90.41	93	1
27.12	96.12	196	2
27.64	96.64	177	2
10.87	79.87	87	0

### Data categorization

A sample of categorized dataset is depicted in Table 3.

**Table 3:** Categorized data

Humidity	Temperature	Step count	Stress level	Stress level categorical
21.33	90.33	123	1	Normal
21.41	90.41	93	1	Normal
27.12	96.12	196	2	High
27.64	96.64	177	2	High
10.87	79.87	87	0	Low

### Data visualization

**Fig. 6:**
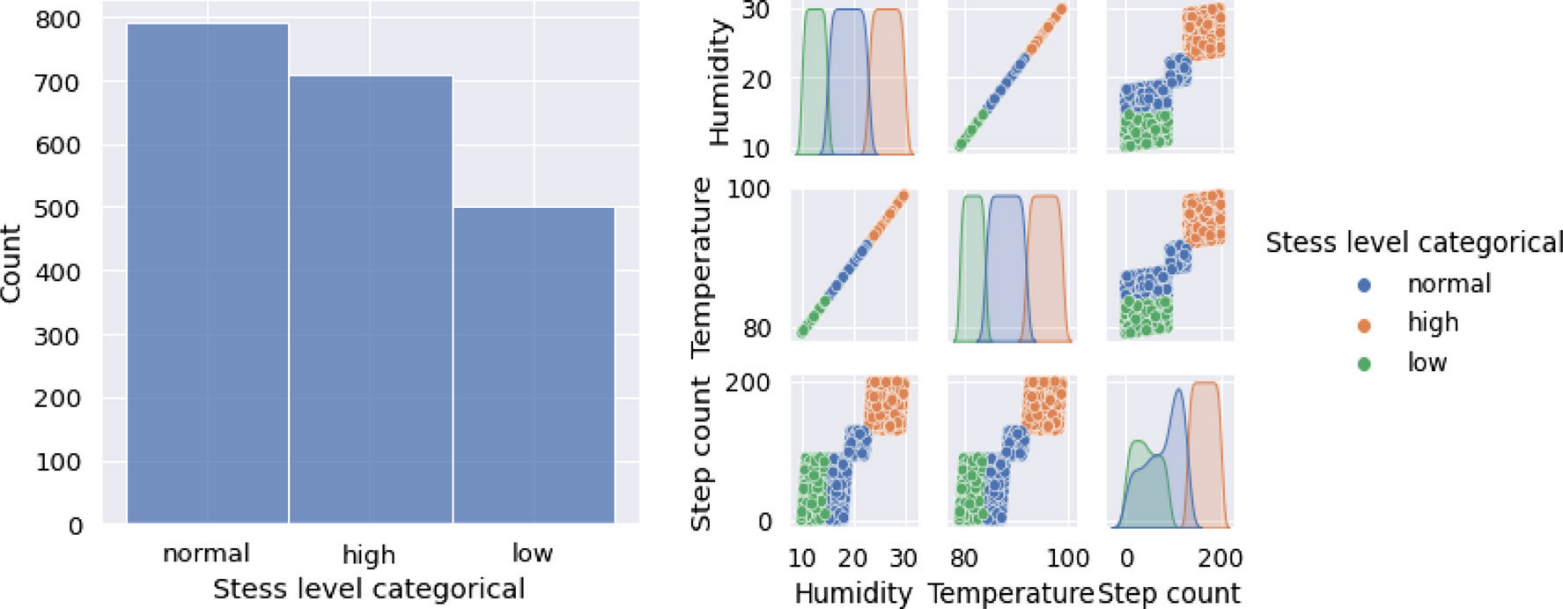
Data visualization

**Fig. 7:**
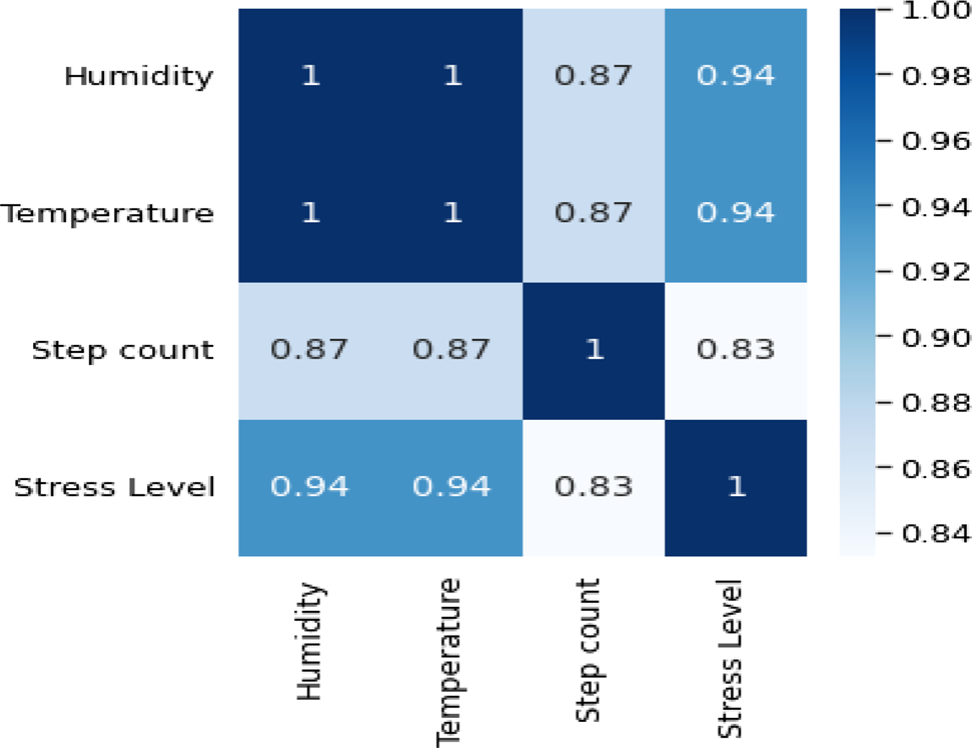
Data heatmap

**Fig. 8:**
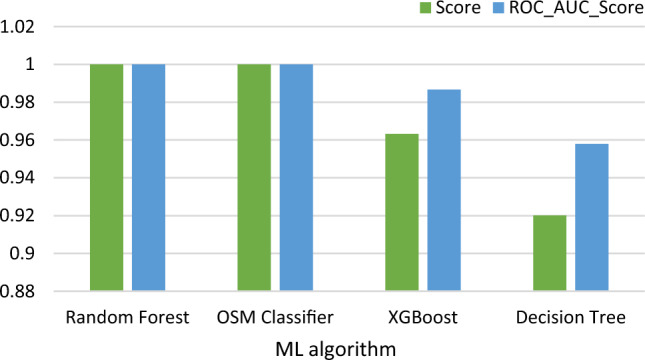
Results of the used ML algorithms

The results of the data visualization phase are illustrated in Fig. 6. The heatmap is shown in Fig. 7. The results for each feature are shown in Table 4. The results of the used ML algorithms are shown in Table 5 and in Fig. 8.

**Table 4:** Results for each feature

	Count	Mean	std	Min	25%	50%	75%
Humidity	2001.0	20.000000	5.777833	10.0	15.0	20.0	25.0
Temperature	2001.0	89.000000	5.777833	79.0	84.0	89.0	94.0
Step count	2001.0	100.141429	58.182948	0.0	50.0	101.0	150.0
Stress level	2001.0	1.104448	0.771094	0.0	0.0	1.0	2.0

**Table 5:** Summarized achieved results of the used machine learning classifiers

Model_after_resampling	Score	ROC_AUC_Score
Random forest	1.000000	1.000000
OSM classifier	1.000000	1.000000
XGBoost	0.963195	0.986748
Decision tree	0.920125	0.957978

**Fig. 9:**
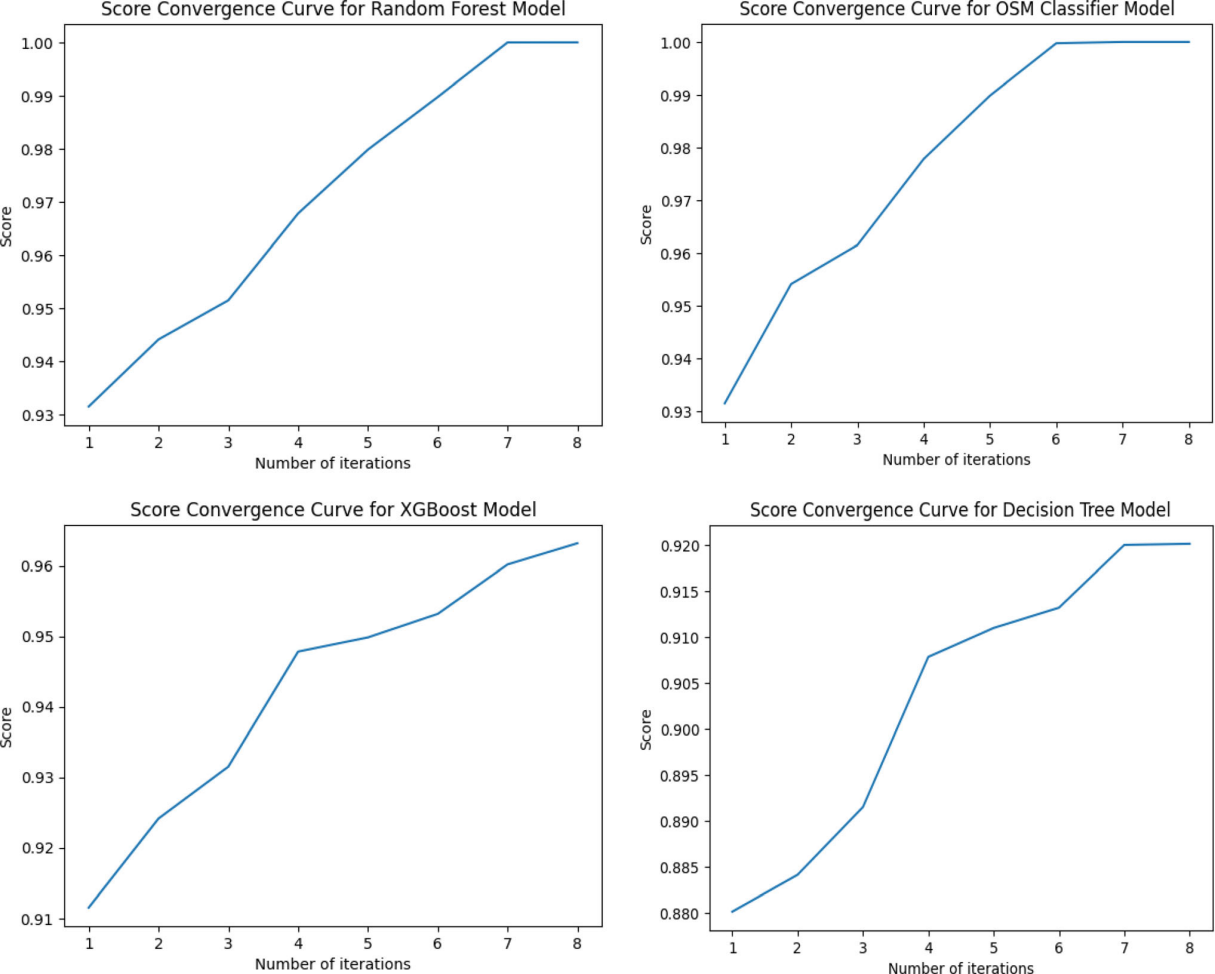
Score convergence curve for each model

The score convergence curve for each model, as depicted in Fig. 9.

From the findings above, it is shown that random forest and OSM are best suited for this classification, with decision tree and XGBoost following closely behind.

The performance of four popular machine learning algorithms (random forest, OSM classifier, decision tree, and XGBoost) was evaluated for stress level detection using data from wearable sensors. The results showed that random forest and OSM classifier performed the best, with perfect accuracy and ROC-AUC scores of 1.0. Decision tree and XGBoost also performed well, but with slightly lower accuracy and ROC-AUC scores.

The success of random forest and OSM classifier in stress level detection can be attributed to their ability to handle large datasets and noisy features, as well as their high accuracy in handling both classification and regression tasks. On the other hand, decision tree and XGBoost are also well-known for their effectiveness in classification tasks but may not perform as well with noisy data.

Overall, the results suggest that machine learning algorithms can effectively detect stress levels using wearable sensor data, with random forest and OSM classifier being the most suitable for this task. These findings can have significant implications in healthcare, as wearable sensor technology continues to become more accessible and may be used to monitor stress levels and other health indicators in real time.

### Results discussion

The results of this study suggest that the proposed stress monitoring algorithm (SMA) using wearable sensors is effective in detecting stress levels in humans. The dataset used in this study was the Stress-Lysis.csv file, which contains 2001 samples of human stress levels based on physical activity, humidity, temperature, and step count.

In the data categorization phase, the data were categorized into three different stress levels: low, normal, and high. This categorization was used to train and test various machine learning classifiers to determine which classifier was most effective in detecting stress levels. The results of the machine learning classifiers were evaluated using the ROC-AUC score, which measures the overall performance of the classifier.

The results of the machine learning classifiers showed that the random forest classifier achieved the highest score, with a perfect score of 1.000 for both the Score and ROC-AUC Score. The OSM classifier also achieved a perfect score of 1.000 for both measures. The XGBoost and decision tree classifiers had lower scores, but still performed well with scores of 0.963 and 0.920 for the Score measure, respectively.

Overall, these findings suggest that the proposed SMA using wearable sensors and machine learning classifiers can be effective in detecting stress levels in humans. The random forest classifier in particular showed excellent performance in this study. However, further research is needed to validate these results on a larger dataset and to determine the applicability of this algorithm in real-world settings. Additionally, the use of other types of sensors, such as heart rate monitors and electroencephalography (EEG) sensors, could be explored to improve the accuracy of stress level detection.

## Conclusion

The study included the uses of IoT in the medical area, the degree to which it is used to enhance conventional practices in various health fields, and the degree to which IoT may raise the standard of healthcare services. The main contributions in this paper are as follows: (1) importing signals from wearable devices, extracting signals from non-signals, performing peak enhancement; (2) processing and analyzing the incoming signals; (3) proposing a new stress monitoring algorithm (SMA) using wearable sensors; (4) comparing between various ML algorithms; (5) the proposed stress monitoring algorithm (SMA) is composed of four main phases: (a) data acquisition phase, (b) data and signal processing phase, (c) prediction phase, and (d) model performance evaluation phase; and (6) grid search is used to find the optimal values for hyperparameters of SVM (C and gamma). From the findings, it is shown that random forest is best suited for this classification, with decision tree and XGBoost following closely behind. The findings of this study have implications for the future of healthcare services, as the use of IoT in medical monitoring can improve the accuracy and efficiency of diagnosis and treatment. Future work can explore other ML algorithms and correlation methods to further improve the performance of the proposed SMA. Additionally, the use of other techniques such as OCNN may also be explored. In the future work, we can use OCNN [41] to achieve better results as it achieved a good performance in [42–48]. We can also use correlation methods like [49].

## Data Availability

https://www.kaggle.com/datasets/laavanya/stress-level-detection.
